# A Novel Opportunity in Minimally Invasive Colorectal Cancer Therapy: Defining a Role for Endoscopic Submucosal Dissection in the United States

**DOI:** 10.1155/2013/681783

**Published:** 2013-11-06

**Authors:** Jonah Cohen

**Affiliations:** Department of Medicine, Beth Israel Deaconess Medical Center and Harvard Medical School, 1st Floor Atrium Suite, 330 Brookline Avenue, Boston, MA 02215, USA

## Abstract

Colorectal cancer is the third most common cancer among both men and women in the United States and the second leading cause of cancer death. Endoscopic submucosal dissection (ESD) is an innovative advanced endoscopic therapy for superficial gastrointestinal neoplasms which is rapidly becoming standard of care particularly in Asia. ESD was first developed for the resection of early gastric cancers; yet ESD for colon tumors has gained increasing attention in recent years. The advantage of ESD over conventional endoscopic resection lies in its potential to achieve en bloc resection regardless of tumor size, leading to more precise histological evaluation and greater potential for cure. Selecting appropriate patients for this procedure involves identifying colorectal cancers with nul risk of lymph node spread. For colorectal ESD to engraft in the United States, the prevalence of such early stage lesions must be defined so that centers of excellence can be developed for high volume clinical practice to offer patients the safest and most efficacious outcomes. This review discusses the endoscopic staging of colorectal neoplasms, indications for colorectal ESD, and the epidemiology of early stage ESD-amenable colorectal cancer in America to better define an opportunity for this important minimally invasive therapy.

## 1. Introduction: Endoscopic Submucosal Dissection and Colorectal Neoplasia

Cancers of the colon and rectum are the third most common cancers among both men and women in the United States and the second leading cause of cancer death [1]. Endoscopic submucosal dissection (ESD) is an innovative advanced endoscopic approach to superficial gastrointestinal neoplasms, which is becoming the standard treatment, particularly in advanced Asian medical centers [2, 3]. ESD was first utilized in the resection of early gastric neoplasms; yet ESD for colon tumors has gained increasing attention in recent years [4–9]. The advantage of ESD over conventional endoscopic mucosal resection is that it has the potential for a high rate of en bloc resection regardless of tumor size, leading to precise histological evaluation of the specimen margins and a lower recurrence rate at long-term followup [5, 10–12]. In one of the largest follow-up studies to date evaluating ESD for colorectal epithelial neoplasms including both adenomas and carcinomas, the 5-year overall/disease-specific survival was greater than 95% [10]. In an analysis of several studies totaling greater than 700 cases of ESD for colorectal cancer, local recurrence rates averaged approximately 1% [7]. Finally, in a recent large-scale multicenter study of long-term outcomes after endoscopic resection for submucosal invasive colorectal cancer, among the patients with low risk features treated by endoscopic resection alone, the 5-year recurrence-free survival and recurrence rates were 98% and 0.8%, respectively [13].

Many experts believe that ESD will someday largely replace colectomy for node-negative colorectal epithelial neoplasia. One recent study in Japan evaluated patients with intramucosal or slightly submucosal invasive colorectal cancer treated with ESD compared with patients who underwent laparoscopic-assisted colectomy for T1 colorectal cancers and concluded that ESD was associated with a lower complication rate and had favorable en bloc and curative resection rates for early cancers with nul risk of lymph node metastasis [2]. In an editorial from *Endoscopy* regarding this study, the author stated: “it is a shame that the vast majority of patients worldwide who have early cancers of the colon and rectum, confined to the mucosa, are subjected to laparoscopic or open colon resections. This is wasteful of healthcare financial resources and really is not optimal care for the patient. There should be an international drive to get surgeons and gastroenterologists up to speed on ESD, so that all patients have access to the “best” treatment for these tumors [14].” Barriers to the adoption of this technique in the United States include greater technical difficulty with a substantial learning curve and longer procedure times, a lack of gastric cancer cases where ESD is easiest and safest to learn, risk of complications such as bleeding and perforation as well as the notable absence of reimbursement guidelines [15]. Selecting appropriate patients for this procedure involves identifying colorectal cancers with nul risk of lymph node spread. Thus, for colorectal ESD to take hold in the United States, the prevalence of such early stage tumors needs to be characterized so that high volume centers of excellence can be developed to offer patients the safest and most efficacious outcomes.

## 2. **Selecting the Appropriate Lesion for ESD: Endoscopic and Pathologic Assessment of Colorectal Neoplasms**

Determining which colorectal neoplastic lesions are amenable to endoscopic resection is a vast topic with much published work on the subject. The Haggitt [16], Paris [17], Vienna [18], and Kudo classification [19] systems are various validated tools for evaluating and predicting the risk of malignancy and invasiveness of various epithelial lesions based on the gross endoscopic appearance, chromoendoscopy, and magnifying endoscopy. Notably, endoscopic ultrasound (EUS) can also be used to increase the predictive value of these classification systems.

Large studies have assessed the prognostic value of these endoscopic staging categories in predicting the risk of submucosal invasion as well as risk of lymph node metastasis [17]. Of note, the mucosa contains three layers from superficial to deep: (1) epithelium with basement membrane, (2) lamina propria, and (3) muscularis mucosae. Thus, the depth of lesion penetration into the submucosal layer is measured as the distance of invasion beyond the muscularis mucosa. The risk of submucosal invasion and subsequent lymph node spread is a central issue in the understanding of early colorectal neoplasia and its subsequent management. Lesions with increased risk of nodal metastasis by current standards require surgical staging which involves lymph node dissection and harvest for pathologic evaluation. Kudo et al. showed in a large series of colorectal neoplasia that depressed-type lesions of 6–10 mm diameter showed submucosal invasion in approximately 24%, as compared with 1.3% in protruding lesions and 0.5% in flat or slightly elevated lesions, and these statistics increased with the size of the lesion [19]. Thus, while for a type 0-I lesion, diameter is a reliable predictive criterion of the risk of submucosal invasion, with type 0-II lesions, the morphologic subtypes have greater importance with the depressed 0-IIc lesions having the greater risk [17]. In these lesions, EUS with high frequency probes at 20 MHz may have an important role. Endoscopy tends to understage superficial lesions, while EUS tends to overstage them, and thus combining these methods is highly predictive of submucosal involvement [20]. Finally, regarding the prevalence of these various lesion subtypes, in a large series of 9533 superficial lesions from a major Japanese center, 57% were 0-I, 39% were 0-IIa,b, and only 4% were 0-IIc [21].

Classification of submucosal invasion is based on the division of the submucosa (sm) into three layers of equivalent thickness, from sm1 to sm3, superficial to deep. Sm1 lesions are further subdivided into three categories (a, b, and c) with regard to the degree of horizontal involvement of the upper submucosal layer. While sm1a + b lesions have a very low risk for metastasis, the malignant potential increases with the depth of submucosal invasion. In addition to the depth of invasion, involvement of the submucosal vessels also portends increased risk of lymph node spread [19]. The risk of nodal metastases has been shown to be high when the invasion reached sm3 near the muscularis propria. On endoscopic mucosal resection (EMR) specimens, the risk of nodal metastasis is nil or small when invasion into the submucosa is less than 1000 micrometers below the muscularis mucosae which corresponds to the sm1 layer [17]. Risks of nodal metastasis by sm layer have been reported as sm1 = <1%, sm2 = 6%, and sm3 = 14% in a study of over 300 “type 0” (superficial polypoid, flat/depressed, or excavated) tumors [17]. One study of 117 submucosal invasive CRCs suggested that when submucosal invasion was less than 850 micrometers in depth and 2500 micrometers in width, there may be no risk of micrometastasis and that EMR alone has complete curative potential in such cases [22]. Additionally, a significant study contributing to the Japanese Society for Cancer of the Colon and Rectum's 2012 guidelines regarding T1-sm1 lesions was derived from a large cohort of 865 patients which found that for nonpedunculated submucosal invasive colorectal carcinomas, the rate of lymph node metastasis was 0% if the submucosal depth was less than 1000 micrometers [23, 24]. Thus, in lesions resected endoscopically with subsequent pathologic evaluation revealing invasion below the sm1 layer or in lesions demonstrating lymphatic invasion, tumor budding, vascular involvement, or poorly differentiated components, additional surgical resection for lymph node staging would likely be recommended which was also corroborated in a recent meta-analysis [25, 26].

Lastly, the tool of magnifying endoscopy in providing an empirical description of the surface pattern of neoplastic lesions can be highly predictive of invasive phenotypes of various lesions which can significantly guide management decisions. Such “pit patterns” have been carefully delineated by Kudo et al. [19]. The noninvasive pit pattern is suggestive of intramucosal neoplasia or submucosal invasion less than 1000 microns which is an appropriate indication for endoscopic treatment. In a large series, histology confirmed this in 98% of 2951 lesions with a noninvasive pattern. The invasive pit pattern, characterized by irregular and distorted epithelial crests, suggests that submucosal invasion is more than 1000 microns. Histology confirmed deep submucosal invasion in 86% of 156 lesions with an invasive pattern [17]. Finally, a recent study importantly assessed the ability of the narrow-band imaging international colorectal endoscopic (NICE) classification to rule out deep submucosal carcinoma invasion with a negative predictive value of 92% [27]. This simplified endoscopic classification system holds promise to facilitate the detection of submucosal involvement which is critical in selecting appropriate patients for ESD.

## 3. Indications for Colorectal ESD**: Consensus Guidelines**

The specific indications for colorectal ESD as recommended by the Colorectal ESD Standardization Implementation Working Group include [8, 28, 29] large-sized (>20 mm in diameter) lesions in which en bloc resection using snare EMR is difficult including nongranular types of lateral spreading tumor (particularly those of the pseudodepressed type), lesions showing V_I_ type pit pattern, carcinoma with submucosal infiltration less than 1000 microns, large depressed-type lesions, and large elevated lesions suspected to be carcinoma. Additional indications for ESD include mucosal lesions with fibrosis related to biopsy, sporadic tumors in chronic inflammation such as in ulcerative colitis, and local residual carcinoma after endoscopic resection that fulfills other aforementioned criteria. An additional indication for ESD often cited includes an adenoma showing a nonlifting sign. As mentioned previously, this evaluation is often determined by endoscopic features, using chromoendoscopy and occasionally magnifying endoscopy or EUS. EUS is used for the unique cases of scarring lesions or when magnifying endoscopy raises the suspicion of massive submucosal invasion. Of note, biopsy is often not needed with adequate chromoendoscopic exam, and, additionally, biopsy can lead to submucosal fibrosis which can lead to increased difficulty and risk in subsequent endoscopic resection [7].

## 4. Colorectal TNM Classification**: A Critical Framework to Guide Therapy**

Another important method for determining the prognosis and management of colorectal neoplasms is the TNM staging system as designated by the American Joint Committee on Cancer (AJCC) [30]. According to the TNM classification for malignant staging, the depth of tumor invasion in the bowel wall corresponds to the T of the classification. Tm (mucosa) and T (in situ) refer to intraepithelial tumors with no invasion of the submucosa. These lesions only involve the mucosa and thus have not grown beyond the muscularis mucosa. As previously mentioned, the mucosa contains three layers from superficial to deep: (1) epithelium with basement membrane, (2) lamina propria, and (3) muscularis mucosae. In T1 lesions, the cancer has grown through the muscularis mucosa and extends into the submucosa. These lesions are sometimes referred to as T1sm. With respect to prevalence of T1 lesions, in a cohort of 7,543 patients who underwent operative treatment for carcinoma of the colon and rectum from 1979 to 1995 at the Mayo Clinic, the incidence of T1 lesions was 8.6 percent; however, the depth of submucosal invasion of these T1 lesions was not apparent [31]. The risk of lymph node metastasis in T1 carcinomas of the colon and rectum ranges from 6 to 14 percent citing several studies; however, these studies did not overtly perform subgroup analyses of the sm1, sm2, and sm3 submucosal layers [31–33]. As notably aforementioned, the risks of nodal metastasis in sm1 (which characterizes lesions that invade less than 1000 micrometers below the muscularis mucosae) have been cited as less than 1% (1/147 patients) [17]. However, as concluded in a recent study on rectal cancer, only the absence of high-grade tumors, invasion of the muscular layer of the intestinal wall, and lymphatic and vascular invasion predicted the success of local excision techniques as radical treatments for rectal cancer [34].

Regarding the TNM colorectal stage classification, stage 0 refers to Tis, N0, and M0 and is often referred to as carcinoma in situ or intramucosal carcinoma. Stage I represents T1-T2, N0, and M0 cancers. In stage I, the cancer invades the submucosa and thus has grown through the muscularis mucosa (T1) or may have grown into the muscularis propria (T2) but involves no lymph nodes. According to the stages defined by the AJCC fifth edition system, 5-year stage-specific survivals were 93.2% for stage I, 82.5% for stage II, 59.5% for stage III, and 8.1% for stage IV [35]. Through the Surveillance, Epidemiology and End Results program (SEER), the National Cancer Institute contracts with nonprofit medical institutions located in specific geographic areas to obtain data on the most invasive and in situ cancer subtypes diagnosed in residents of the 12 SEER geographic areas which collectively cover about 14% of the total US population. The SEER program follows all previously diagnosed patients on an annual basis to calculate observed and relative survival rates [36]. Between 1988 and 2001, the SEER database reported 247,671 cases of colorectal cancer. After all exclusions, 182,589 cases were evaluated in the SEER statistics (male = 92,880, female = 89,709, white = 150,522, black = 16,830, and other = 15,327). A total of 11,041 carcinoma in situ cases were excluded from the analysis. It is unclear why stages 0 and 1 are included as one category and it is also ambiguous why there were 11,041 carcinoma in situ (CIS) patients not included in the published analysis; however, given that stage 0 is often synonymous with CIS, there appear to be many CIS cases that are ultimately included in the 182,589 patients. Of the 182,589 cases, 26.3% were classified as stage 0/1. Stage 0/I colorectal cancers were further subdivided into the depth of penetration into the wall based on SEER extent of disease (EOD) extension codes. While in situ lesions were excluded from the published SEER analysis, the AJCC considers invasion of the lamina propria to be equivalent to in situ or noninvasive disease. Thus, while cancers which meet these criteria are considered to be malignant neoplasms, with respect to AJCC stage they are classified as stage 0. Thus, by this AJCC staging report, stage 0 is limited only to those patients whose tumor had extended to the lamina propria. Given that all stage 0 and some stage 1 colorectal cancers are theoretically amenable to endoscopic treatments (ESD and potentially EMR), further analysis of this data could be valuable with respect to determining the prevalence of endoscopically treatable colorectal cancer in the United States. In turn, this information could assist in establishing the need for ESD centers of excellence in America. It is important to note that large adenomatous polyps represent another significant category of lesions where ESD may have a role in reducing rates of local recurrence compared to other conventional endoscopic resection techniques. ESD may also provide an organ-sparing alternative for adenomatous polyps which have been traditionally removed by surgery. Lastly, given the aging demographics in our country, the burden of colorectal cancers will likely increase further inviting development of minimally invasive methods such as ESD to treat these malignancies.

## 5. Conclusions**: A Novel Opportunity in Minimally Invasive Colorectal Cancer Therapy**

ESD is an innovative advanced endoscopic approach to superficial gastrointestinal neoplasms which is increasingly becoming a standard treatment particularly in Asian medical centers and has the potential to revolutionize treatment of early alimentary cancers in America as well. Colorectal cancer represents an important potential niche for clinical application of ESD in the United States given the prevalence of these tumors. Given the technical difficulty of ESD, further ex vivo and in vivo training programs must be developed to better define the learning curve for safe and effective colorectal ESD. Additionally, reimbursement guidelines will need to be created which address the time-consuming nature and expert training required for this minimally invasive procedure. Further, in an era of increasing fiscal responsibility, it is important to note that recent evidence suggests that utilizing ESD for treatment of colorectal cancer may also reduce costs compared with conventional surgical therapies [2]. Careful patient selection will be critical to successful ESD in identifying patients' tumors with nul risk of lymph node metastasis, necessitating additional training for US endoscopists in chromoendoscopy and Kudo/Paris preoperative tumor classifications. Finally, for colorectal ESD to engraft in the United States, the prevalence of early colorectal cancers (stage 0 and stage 1, sm1) must be defined so that centers of excellence can be developed for high volume clinical practice to offer patients the safest and most efficacious outcomes. However, an important question remains as to whether the biology of colon cancer in Asia may differ with respect to the prevalence of lateral spreading cancers. Further studies are needed to clarify the epidemiology of early stage ESD-amenable colorectal cancer in America to better define a role for this important organ-sparing alternative to surgery.
